# Oscillometric blood pressure values in infants at 3, 6 and 12 months of age: a cohort study

**DOI:** 10.1136/bmjpo-2026-004761

**Published:** 2026-06-17

**Authors:** Elin Ørbeck Vallevik, Mads Nikolaj Holten-Andersen, Karin C Lødrup Carlsen, Håvard Ove Skjerven, Riyas Vettukattil, Corina Silvia Rueegg, Eva Maria Rehbinder, Björn Nordlund, Cilla Söderhäll, Gunilla Hedlin, Guttorm Nils Haugen, Anne Cathrine Staff, Morten Nilsen, Henrik Holmstrøm

**Affiliations:** 1 Department of Paediatric and Adolescent Medicine, Innlandet Hospital Trust, Lillehammer, Norway; 2 Institute of Clinical Medicine, University of Oslo Faculty of Medicine, Oslo, Norway; 3 Division of Paediatric and Adolescent Medicine, Oslo University Hospital, Oslo, Norway; 4 Oslo Centre for Biostatistics and Epidemiology, Oslo University Hospital, Oslo, Norway; 5 Epidemiology, Biostatistics and Prevention Institute, University of Zurich, Zürich, Switzerland; 6 Department of Dermatology, Oslo University Hospital, Oslo, Norway; 7 Department of Women's and Children’s health, Karolinska Institutet, Stockholm, Sweden; 8 Astrid Lindgrens Childrens Hospital, Karolinska University Hospital, Stockholm, Sweden; 9 Division of Gynaecology and Obstetrics, Oslo University Hospital, Oslo, Norway; 10 Department of Chemistry Biotechnology and Food Science, Norwegian University of Life Sciences, As, Norway

**Keywords:** Nephrology, Cardiology

## Abstract

**Objective:**

To determine reference blood pressure (BP) values in a cohort of healthy infants at 3, 6 and 12 months of age.

**Design:**

An antenatally recruited population-based mother–child cohort study.

**Setting:**

The multicentre Preventing Atopic Dermatitis and ALLergies in children study, recruiting non-selected pregnant women in Norway and Sweden between 2014 and 2016. Infant oscillometric BP was measured at clinical follow-up visits at 3, 6 and 12 months of age.

**Patients:**

In total, 2100 infants had BP readings available during infancy.

**Main outcome measures:**

Systolic, diastolic and mean arterial BP at each follow-up visit based on successful BP measurements defined as three readings obtained in a calm state with systolic BP within a 20 mm Hg range.

**Results:**

Successful BP measurements were obtained in 1778 infants at 3, 6 and/or 12 months of age. Median systolic/diastolic BP (mean arterial pressure) was 95/56 (69) mm Hg at age 3 months, 96/57 (72) mm Hg at 6 months and 95/57 (71) mm Hg at 12 months.

**Conclusions:**

This study establishes reference values for oscillometric BP in healthy infants and shows consistent measurements across 3, 6 and 12 months of age.

**WHAT IS ALREADY KNOWN ON THIS TOPIC**

- While oscillometric measurements are commonly used in clinical practice, normative blood pressure values for infants beyond the neonatal period remain limited.

**WHAT THIS STUDY ADDS**

- This study provides reference blood pressure values for infants at 3, 6 and 12 months of age, derived from standardised oscillometric measurements in a large, prospective, population-based cohort.

**HOW THIS STUDY MIGHT AFFECT RESEARCH, PRACTICE OR POLICY**

- These reference values may support recognition of abnormal blood pressure in infancy and guide clinical decision-making and follow-up.

## Introduction

 Normal values are essential for the correct evaluation of blood pressure (BP). The most widely used nomograms for evaluating infant BP originate from the American Second Task Force on Blood Pressure Control in Children from 1987.^1^ Although the guidelines from the American Academy of Pediatrics have been updated several times (in 1996, 2004 and 2017),^2^,^4^ the normative BP values for children during their first year of life remain based on the 1987 data, due to the lack of large-scale studies on infant BP.^5^ The BPs in the Second Task Force report were derived from Doppler (systolic BP) and mercury sphygmomanometer (diastolic BP) measurements conducted in four studies during the 1970s and 1980s.^6^,^9^ A systematic review from 2015 by Cantinotti *et al* states that BP data on children from 0 to 12 months are extremely limited.^10^

Today, the automatic oscillometric method is widely used for measuring BP and is considered suitable and feasible for initial BP screening in the paediatric population, as it is convenient and easy to use, with less dependence on the person performing the measurement compared with auscultatory methods.^11 12^ To our knowledge, only four studies have provided BP data for infants after the newborn period, utilising an oscillometric device, with sample sizes ranging from 118 to 430 infants between 1 and 12 months of age.^13^,^16^ Two of these studies^13 15^ date back more than 35 years. When evidence-based knowledge is lacking, clinical decisions often rely on a broad consensus among experienced clinicians. However, a survey conducted across 23 Nordic University hospitals in 2017 revealed significant discrepancies among experienced paediatricians regarding the assumed normal range of infant BP.^17^ This underscores the need for comprehensive normal BP values from automatic oscillometric devices, based on large populations of healthy infants.

The primary aim of this study was to determine oscillometric BP values in a general population at 3, 6 and 12 months of age. The secondary aim was to explore potential differences in BP in boys and girls from 3 to 12 months of age. Further aims were to explore the potential impact on BP values of arousal state, and the number and sequence of measurements.

## Methods

### Study design and population

The present study originates from the prospective antenatally recruited mother–child birth cohort, the Preventing Atopic Dermatitis and ALLergies in children (PreventADALL) study, described in more detail elsewhere.^18 19^ Briefly, 2697 pregnant women were recruited at the routine 18-week fetal ultrasound examination from the general populations in Norway (Oslo and Østfold) and Sweden (Stockholm) between 2014 and 2016. Maternal inclusion criteria were mono pregnancies or twin pregnancies between 16 and 22 weeks of gestational age (GA), sufficient language skills to follow the study procedures and no plans to move outside the study areas within 1-year postpartum. Participants were excluded in case of severe maternal or fetal disease. In all, 2397 infants born to the participating women were included at birth provided a GA ≥35.0 weeks and no severe illness; none was excluded for having potential risk factors for hypertension. Consent was later withdrawn for three infants.

**Figure 1:**
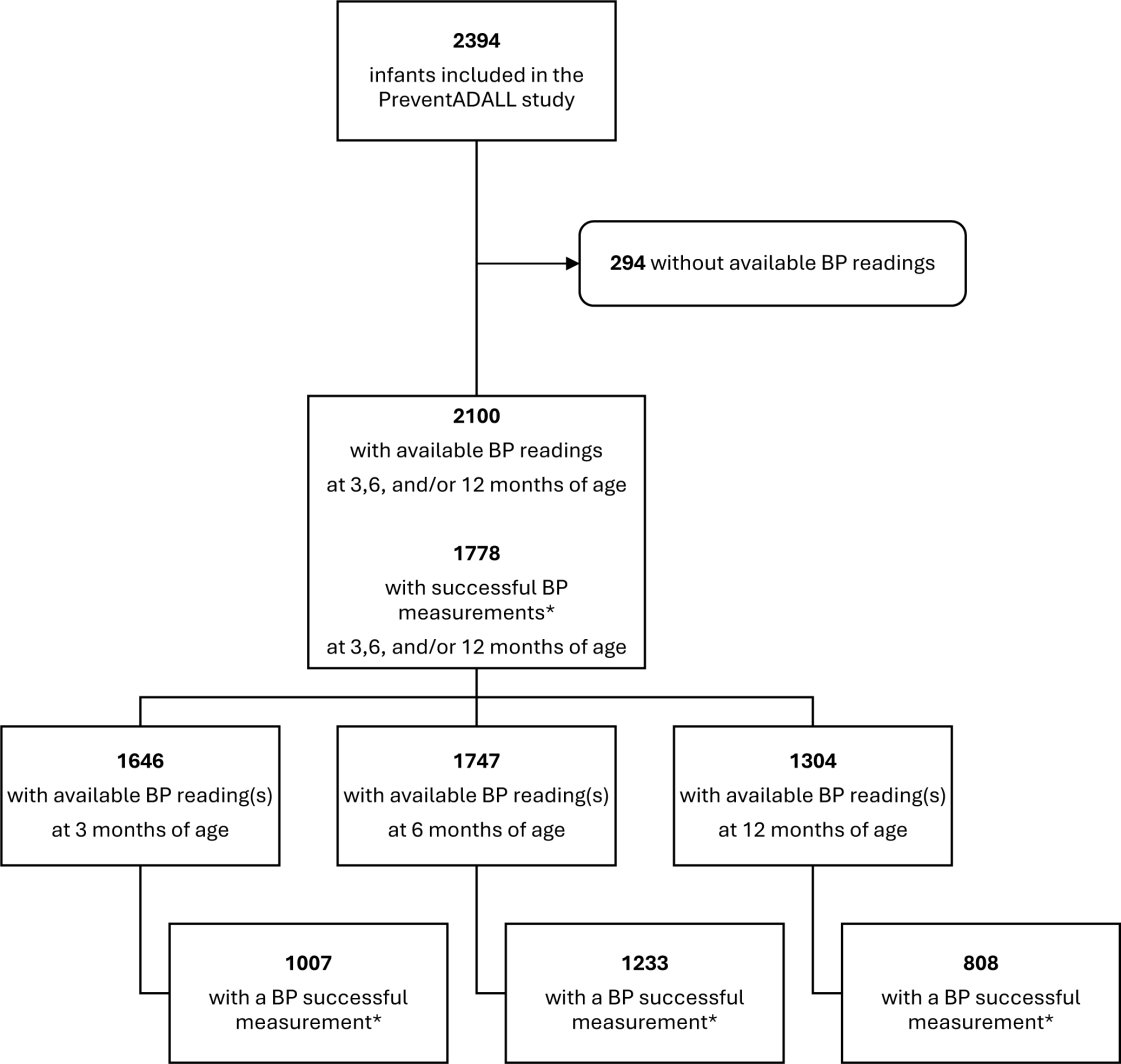
Study flow chart. *A successful measurement is defined by three available blood pressure readings obtained in a calm state and a systolic blood pressure range of maximum 20 mm Hg. BP, blood pressure.

The infants attended clinical follow-up examinations at 3, 6 and 12 months of age, which included standardised oscillometric BP measurements. The present study included all infants with at least one available BP reading at any of these ages (figure 1).

### Procedures and outcomes

BP measurements were obtained at 3, 6 and 12 months of age by trained study personnel following a standard operating procedure (see online supplemental material for the detailed procedure). The procedure was explicitly designed to generate BP reference data, and strict adherence to it was ensured across all study sites. The GE Carescape Dinamap V.100,^20^ an automatic oscillometric BP monitor, was utilised.^21^ One of two cuffs was applied to the right upper arm depending on the arm’s length and circumference; GE Critikon 2525 Neonatal number 5 (8–15 cm)^22^ or GE Critikon Dura-Cuf Ref 2201 (12–19 cm).^23^ Arm circumference was measured at the midpoint with a tape, and the cuff with the marked range that best matched the measurement was selected, avoiding cuffs that extended over a joint. Measurements were made with infants in a supine position at 3 and 6 months and in a sitting position at 12 months of age, reflecting standard clinical practice and feasibility at these ages. For each reading, the infant’s state of arousal was assessed by trained study personnel using predefined clinical criteria. Agitation was recorded if the infant showed active movement, struggling or crying, whereas a calm state was defined as lying or sitting quietly without movement or signs of arousal.

The goal was a successful BP measurement at each age consisting of three BP readings with systolic BP within a range of 20 mm Hg, obtained while the infant was calm. In case of variation in systolic BP readings >20 mm Hg and/or the infant being agitated, readings were repeated. If three readings within 20 mm Hg were not obtained after three additional attempts, we recorded the first three results (at 3 and 6 months) or the three results with highest correspondence (at 12 months).

The primary outcomes were systolic BP, diastolic BP and mean arterial pressure (MAP), in line with successful measurements. Secondary outcomes were differences in BP values between boys and girls at 3, 6 and 12 months of age, and differences in BP values obtained in a calm versus agitated state, for infants with readings in both states.

### Statistical analysis

Normative BP values are provided with the primary outcomes based on all available successful measurements at 3, 6 and 12 months of age. The mean of the three readings for each measurement was used in the analysis. The BP values were normally distributed and are reported as mean, SD, percentiles and z-scores, overall and for boys and girls separately.

Potential differences in BP values by age and between boys and girls were assessed in all infants with at least one successful measurement during infancy, using a linear mixed effects model for repeated measures, including a Time×Sex interaction term. The model uses all available data and accounts for missing observations under a maximum likelihood framework, assuming that data are missing at random. Given the close relationship between age and weight during infancy, weight was not included in the mixed model analysis. The aim was to assess differences in BP development between boys and girls over time. In this context, weight is likely to lie on the causal pathway and was, therefore, not included to avoid overadjustment. No additional covariates were included in the model as the analysis focused on describing trajectory differences rather than isolating independent effects of specific variables.

The effect of arousal state was assessed by paired t-tests comparing BP readings in the agitated versus calm state in infants with at least one reading in each state. If more than one reading in a state was available, the mean of available readings was used in the analysis.

To assess the potential role of number and sequence of readings for establishing an infant’s BP, among those with at least one successful measurement, we report the mean, SD and percentiles for various combinations of readings: the mean of three readings, the mean of the two closest readings, the mean of the first two readings, the mean of the last two readings and the first reading alone at 3, 6 and 12 months of age. Additionally, we report the mean and SD of the maximum difference between the highest and lowest BP readings in each scenario.

Statistical analyses were conducted using STATA/SE V.18.0. No adjustment for multiplicity was performed because the study’s primary aim was of descriptive nature. A p value <0.05 was considered statistically significant. Descriptive statistics and t-tests were based on available data without imputation of missing data (complete case analysis).

### Patient and public involvement

Patients or the public were not involved in the design, conduct, reporting or dissemination plans of this research.

## Results

Of the 2394 eligible infants, 2100 had BP readings available at 3, 6 and/or 12 months of age and 1778 met the criteria for a successful measurement at least once (figure 1). Most infants were of Nordic ethnicity. Baseline characteristics at inclusion and anthropometric measurements at follow-up were similar between the infants with and without successful BP measurements, summarised in table 1 and in online supplemental table 1.

**Table 1:** Baseline characteristics of infants with and without successful blood pressure measurements*

Baseline characteristics	Infants with successful measurements (N=1778)	Infants without successful measurements (N=322)
Male sex	953 (54)	161 (50)
Gestational age at birth, days†	281 (9)	279 (10)
Birth weight, g†	3583 (482)	3505 (483)
Birth length, cm†	51 (2)	50 (2)
Age mother, years†	33 (4)	32 (4)
Age father, years†	35 (5)	35 (5)
Weight of mother at inclusion, kg†	70 (11)	72 (12)
BMI of mother at inclusion, kg/m^2^†	24.6 (3.4)	25.7 (4.5)
Delivery method		
Vaginal	1490 (84)	267 (83)
Caesarean section	286 (16)	54 (17)
Missing	2 (0.1)	1 (0.3)
Maternal country of origin		
Nordic country	1467 (83)	271 (84)
Other	150 (8)	28 (9)
Missing	161 (9)	23 (7)
Paternal country of origin		
Nordic country	1423 (80)	264 (82)
Other	159 (9)	27 (8)
Missing	196 (11)	31 (10)
Maternal education		
Preliminary or high school only‡ (9–13 years of schooling)	149 (8)	44 (14)
Higher education for <4 years	483 (27)	100 (31)
Higher education for ≥4 years	976 (55)	153 (48)
Other	2 (0.1)	0 (0)
Missing	168 (9)	25 (8)
Partner education		
Preliminary or high school only‡ (9–13 years of schooling)	270 (15)	76 (24)
Higher education for <4 years	478 (27)	77 (24)
Higher education for >=4 years	787 (44)	132 (41)
Other	16 (1)	5 (1.6)
Missing	227 (13)	32 (10)

Data are n (%) or mean (SD). Percentages may not sum to 100 because of rounding.

* A successful measurement is defined by three available blood pressure readings obtained in a calm state and a systolic blood pressure range of maximum 20 mm Hg.

† Missing observations for continuous variables for infants with/without successful measurements: gestational age at birth 4/2. Birth weight 7/2. Birth length 91/21. Age mother 0/0. Age father 269/42. Weight mother at inclusion 20/4. BMI of mother at inclusion 29/5.

‡ Preliminary or high school only refers to 9–13 years of schooling.

BMI, body mass index.

**Figure 2:**
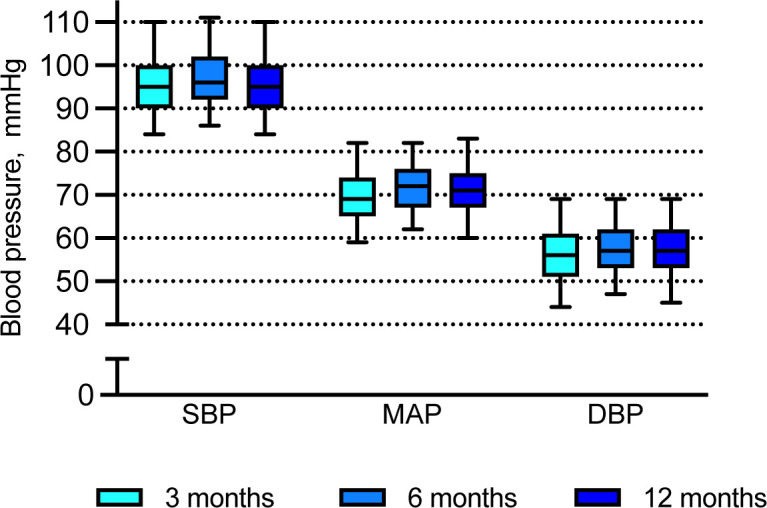
Normative blood pressures at 3 (N=1007), 6 (N=1233) and 12 (N=808) months of age. Each box represents the IQR, the line inside marks the median and the whiskers represent the 5th and 95th percentiles. Including infants with successful measurements at each time point. A successful measurement is defined by three available blood pressure readings obtained in a calm state and a SBP range of maximum 20 mm Hg. DBP, diastolic blood pressure; MAP, mean arterial pressure; SBP, systolic blood pressure.

At 3, 6 and 12 months of age, the median systolic/diastolic BP for infants with successful measurements were 95/56, 96/57 and 95/57 mm Hg, and the corresponding median MAP values were 69, 72 and 71 mm Hg (table 2A and figure 2). The ranges between the 5th and 95th percentiles were 26, 25 and 26 mm Hg for the systolic BP at 3, 6 and 12 months of age, and the corresponding ranges for diastolic BP were 25, 22 and 24 mm Hg and for MAP 23, 20 and 23 mm Hg. Table 2B shows the z-scores from −2 to 2.

**Table 2:** Normative blood pressures at 3, 6 and 12 months of age

A	3 months			6 months			12 months		
	All (N=1007)	Boys (N=553)	Girls (N=454)	All (N=1233)	Boys (N=655)	Girls (N=578)	All (N=808)	Boys (N=451)	Girls (N=357)
Systolic blood pressure, mm Hg									
Mean (SD)	95 (8)	96 (8)	94 (8)	97 (8)	98 (8)	96 (7)	96 (8)	96 (8)	95 (8)
p5	84	84	83	86	86	86	84	83	84
p25	90	91	88	92	93	91	90	91	90
p50	95	96	93	96	97	96	95	95	94
p75	100	101	98	102	103	101	100	100	100
p95	110	110	108	111	112	110	110	111	108
Diastolic blood pressure, mm Hg									
Mean (SD)	56 (7)	57 (7)	55 (7)	57 (7)	58 (7)	57 (7)	57 (7)	58 (7)	57 (7)
p5	44	44	44	47	47	46	45	45	45
p25	51	51	50	53	53	52	53	53	53
p50	56	56	55	57	58	57	57	58	57
p75	61	61	60	62	63	61	62	62	62
p95	69	69	68	69	70	68	69	68	69
Mean arterial pressure, mm Hg									
Mean (SD)	69 (7)	70 (7)	68 (7)	72 (6)	72 (6)	71 (6)	71 (7)	71 (7)	71 (7)
p5	59	59	58	62	63	61	60	60	60
p25	65	65	64	67	68	67	67	67	67
p50	69	70	68	72	72	71	71	71	71
p75	74	75	72	76	77	75	75	76	75
p95	82	82	81	82	84	81	83	83	82

B	3 months			6 months			12 months		
	All (N=1007)	Boys (N=553)	Girls (N=454)	All (N=1233)	Boys (N=655)	Girls (N=578)	All (N=808)	Boys (N=451)	Girls (N=357)
Systolic blood pressure, mm Hg									
Mean (SD)	95 (8)	96 (8)	94 (8)	97 (8)	98 (8)	96 (7)	96 (8)	96 (8)	95 (8)
Z −2	79	80	78	81	82	82	80	80	79
Z −1	87	88	86	89	90	89	88	88	87
Z 0	95	96	94	97	98	96	96	96	95
Z 1	103	104	102	105	106	103	104	104	103
Z 2	111	112	110	113	114	110	112	112	111
Diastolic blood pressure, mm Hg									
Mean (SD)	56 (7)	57 (7)	55 (7)	57 (7)	58 (7)	57 (7)	57 (7)	58 (7)	57 (7)
Z −2	42	43	41	43	44	43	43	44	43
Z −1	49	50	48	50	51	50	50	51	50
Z 0	56	57	55	57	58	57	57	58	57
Z 1	63	64	62	64	65	64	64	65	64
Z 2	70	71	69	71	72	71	71	72	71
Mean arterial pressure, mm Hg									
Mean (SD)	69 (7)	70 (7)	68 (7)	72 (6)	72 (6)	71 (6)	71 (7)	71 (7)	71 (7)
Z −2	55	56	54	60	60	59	57	57	57
Z −1	62	63	61	66	66	65	64	64	64
Z 0	69	70	68	72	72	71	71	71	71
Z 1	76	77	75	78	78	77	78	78	78
Z 2	83	84	82	84	84	83	85	85	85

Normative systolic, diastolic and mean arterial blood pressures at 3, 6 and 12 months of age are presented as percentiles (A) and z-scores (B), overall and for boys and girls separately. Including infants with successful measurements at each time point. A successful measurement is defined by three available blood pressure readings obtained in a calm state and a systolic blood pressure range of maximum 20 mm Hg.

p, percentile; Z, z-score.

**Figure 3:**
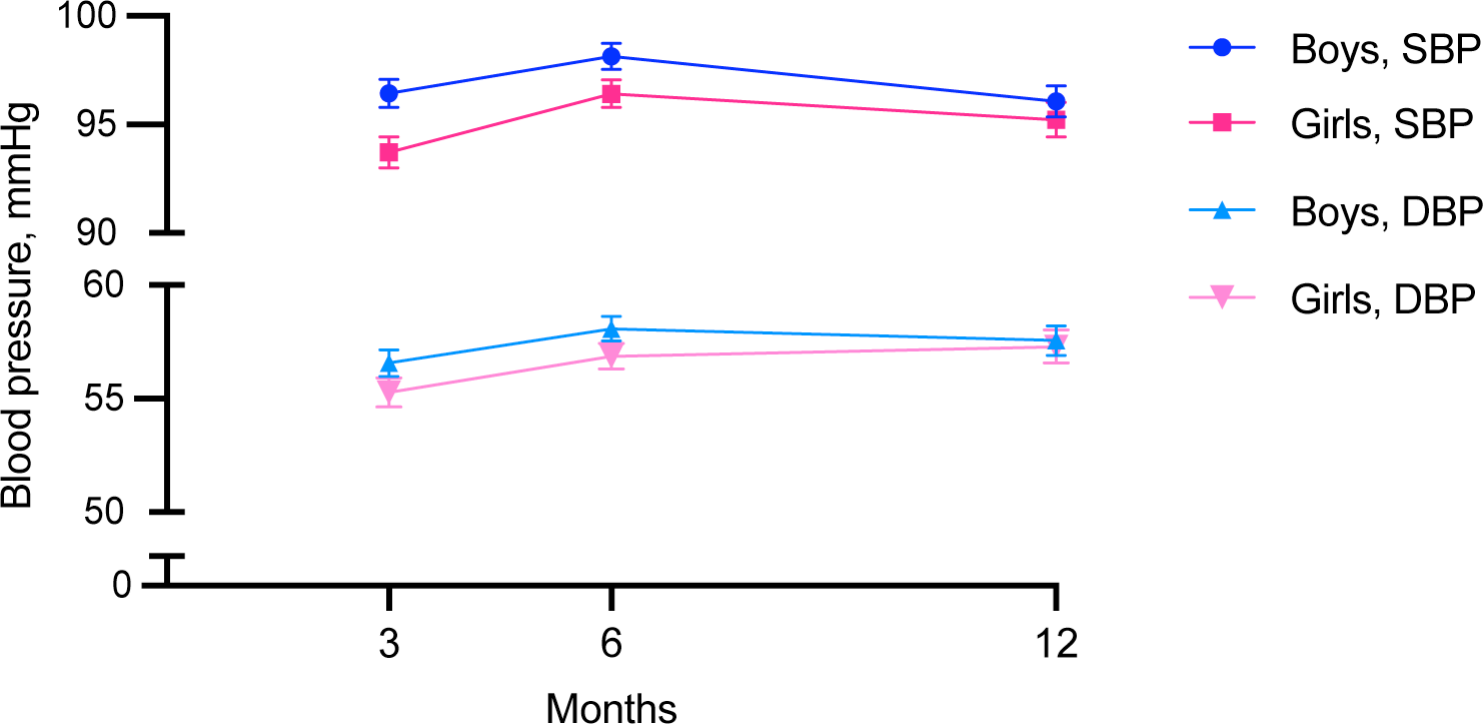
Changes in blood pressure over the first year of life for boys and girls, from linear mixed regression model for repeated measures. The symbols represent the predictive means with 95% CIs. Including infants with successful measurements on at least one time point. A successful measurement is defined by three available blood pressure readings obtained in a calm state and a SBP range of maximum 20 mm Hg. DBP, diastolic blood pressure; SBP, systolic blood pressure.

Boys had higher mean systolic BP, diastolic BP and MAP than girls at 3 and 6 months of age, but no sex differences were observed at 12 months (figure 3, table 2A,B). Compared with boys at 3 and 6 months, the difference in mean systolic BP in girls was −2.7 (95% CI −3.7 to −1.8) and −1.7 mm Hg (95% CI −2.6 to −0.9), the difference in mean diastolic BP was −1.3 (95% CI −2.3 to −0.4) and −1.2 mm Hg (95% CI −2.0 to −0.4) and the difference in mean MAP were −1.9 (95% CI −2.7 to −1.1) and −1.6 mm Hg (95% CI −2.3 to −0.9) (figure 3).

Readings in both calm and agitated states were available in 79 infants at 3 months, 150 at 6 months and 139 at 12 months of age. The BPs were significantly higher in the agitated state across all three ages. The mean difference for the systolic BP was 9.9 mm Hg (95% CI 6.7 to 13.0) at 3 months, 11.1 mm Hg (95% CI 9.2 to 12.9) at 6 months and 7.8 mm Hg (95% CI 5.8 to 9.9) at 12 months of age. For diastolic BP, the mean differences at 3, 6 and 12 months of age were 8.6 mm Hg (95% CI 5.6 to 11.6), 5.8 mm Hg (95% CI 4.1 to 7.4) and 3.2 mm Hg (95% CI 1.5 to 4.8), and for MAP 9.3 mm Hg (95% CI 6.7 to 12.1), 6.6 mm Hg (95% CI 5.1 to 8.2) and 4.7 mm Hg (95% CI 3.2 to 6.2) (online supplemental table 2).

The mean and median BP values in infants with successful measurements were largely similar at all ages, regardless of whether we used the first reading alone, the mean of three readings or the mean of the first two, the last two, or the two closest readings (online supplemental table 3).

## Discussion

In this large cohort of non-selected infants, BP values remained consistent at 3, 6 and 12 months of age, with median systolic/diastolic BPs of 95/56, 96/57 and 95/57 mm Hg. Although BP values were higher in boys than girls at 3 and 6 months of age, the differences were negligible and absent by 12 months. BP values were higher in agitated compared with calm states. In calm infants, the first reading was similar to the mean BP of various combinations of two or three BP readings.

To the best of our knowledge, this is the largest study to provide normative oscillometric BP values in infancy after the newborn period. Our median BP values were on average 5 mm Hg higher than those reported in the American Second Task Force nomograms.^1^ However, the 95th percentiles align closely, despite the differences in measuring methods.

Over the past 35 years, only two other studies, to our knowledge, have utilised an oscillometric method to establish normative BP values for infants under 12 months of age, both based on smaller, single-centre samples.^14 16^ Kent *et al*^14^ included 150 infants at 6 months and 118 infants at 12 months of age, and reported higher BP values than in our cohort, with median systolic/diastolic BP (MAP) of 102/62.5 (75) mm Hg at 6 months and 101/64 (75) at 12 months. Notably, they reported a 95th percentile of 120 mm Hg for systolic BP, despite excluding potential confounders for infant hypertension, such as maternal diabetes and hypertension. Different measuring devices, our larger study population and our exclusion of agitated infants might explain the discrepancies. A recent Spanish study by Blanca-Jover *et al*^16^ included 228 infants, 60% with minor cardiac diagnoses and reported similar oscillometric BP values to ours in the subset of 136 infants aged 3–12 months.

In line with our findings at 12 months of age, Al Salloum *et al*^24^ reported oscillometric BP medians and 95th percentiles of 93/57 and 110/72 mm Hg for 1171 1-year-old Saudi children, although the exact age in months is unclear.

Studies using the auscultation method have reported lower BPs for infants than our study, exemplified with median values at 3 months of age of 88/52^25^ and 81/41.^26^ This aligns with the findings of Blanca-Jover *et al* and with studies in older children showing discrepancies between BP measurements obtained using oscillometric and manual methods.^12 16 27 28^ Also, oscillometric BP readings may vary between devices due to device-specific algorithms.^12^ Standardised normative values are needed for each measurement method. In this study, the same device was used across all three centres to ensure consistent measurements, providing reliable oscillometric BP reference values for infants aged 3–12 months, within the context of the device used.

Compared with percentiles, z-scores are statistically more robust, provide greater precision at extreme values and facilitate longitudinal assessment. Given their increasing use in paediatrics, z-scores were reported in this study to support both clinical practice and future paediatric BP research.^29 30^

The higher BP observed in boys compared with girls at 3 and 6, but not at 12 months of age, was statistically significant, but the differences were small (1.2 to 2.7 mm Hg). We, therefore, consider these slight and transient differences unlikely to be of major clinical importance. Most studies and guidelines stratify BP values by sex, although the differences between infant boys and girls are small, often just a few mm Hg or none at all.^414 16 24^,^26^ With BP levels showing minimal variation from 3 to 12 months of age and negligible sex differences, we suggest using unified normative values for all infants in this age range: 5th percentiles for systolic BP/diastolic BP (MAP) of 85/45 (60) mm Hg; 50th percentiles of 95/57 (70) mm Hg; and 95th percentiles of 110/69 (83) mm Hg, respectively.

Most BP guidelines^4 31 32^ use the 95th percentile as a cut-off for hypertension and recommend that a high oscillometric BP in infants should be confirmed by auscultation. Thus, we emphasise the need for further careful clinical assessment before diagnosing hypertension in infants with an oscillometric BP measurement above the 95th percentile.

Although the BP values in our study were generally stable between 3 and 12 months of age, a modest decline is apparent at 12 months (most evident in figure 3). This pattern has also been described by Dionne *et al*.^5^ One plausible explanation is the change in infant positioning from supine to sitting at 12 months. This positional effect should be accounted for when applying these normative values in clinical practice.

To the best of our knowledge, this study is unique in recording and reporting the arousal state of the infants during every oscillometric BP reading. The significantly higher BP when the infant is agitated emphasises the importance of creating a calm environment to ensure reliable measurements.

In calm infants, the BP values were consistent whether based on the first reading alone or various combinations of the subsequent two or three readings. This consistency also held when compared with the mean of the calm readings in the subgroup of infants with readings in both calm and agitated states, exemplified with mean systolic BPs of 95 mm Hg at 3 months, 98 mm Hg at 6 months and 97 mm Hg at 12 months of age in this subgroup (online supplemental table 2). In line with our findings, Sarici *et al* concluded that a single BP reading is sufficient for term-born newborns (N=138) when free from struggling, crying or movement, rather than averaging repeated readings.^33^ Most guidelines recommend obtaining multiple BP readings during a clinical visit, due to the natural variability of BP.^4 31 32^ However, these recommendations are mostly based on studies in adults and older children.^34^,^36^ Our findings suggest that for infants aged 3–12 months, a single BP reading is reliable as long as the infant is calm, and it is more accurate than measurements that include repeated readings in an agitated state. This is an encouraging result in a busy clinical setting. For infants who cannot be assessed calmly, repeated measurements and clinical judgement remain necessary.

Strengths of our study include its large, population-representative cohort, a longitudinal design and adherence to standardised protocols focusing on calm infants. We anticipate that these results will serve as a valuable clinical tool for assessing infant BP, thereby filling a significant gap in the existing knowledge.

Our study’s limitations include a predominantly Nordic infant population, preventing us from addressing potential ethnic and regional BP variations. Additionally, our results are specific to one type of oscillometric device. It is important to note that each manufacturer uses different computational algorithms, leading to potential variability in measurements across different devices.^37 38^ The device used in our study has been validated for infants, but the supporting validation study has notable limitations: it included only 15 neonates (with calf cuff placement) and 6 older infants, was not an independent validation, and is available only as an abstract with limited details.^21^ Overall, validation studies of oscillometric BP devices in healthy infants after the newborn period up to 12 months of age are limited, posing a challenge both for clinical practice and for research on BP in this age group. Nonetheless, current American,^4^ Canadian^32^ and European^31^ guidelines state that oscillometric measurements may be used for BP screening in infants, and the device used in this study is commonly used in clinical practice worldwide, providing a need for reliable reference BP values from this device.

In conclusion, this study found consistent oscillometric BP values across 3, 6 and 12 months of age in a large population of healthy infants. While boys showed slightly higher values than girls at 3 and 6 months, the differences were minimal and not present at 12 months of age. Thus, the same normative oscillometric BP values can be applied to all infants aged 3–12 months. Additionally, a single BP reading in a calm infant is more reliable than measurements including repeated readings during agitation.

## Supplementary material

10.1136/bmjpo-2026-004761 online supplemental file 1

## Data Availability

Data are available upon reasonable request.
