# Supplementary material for: Oscillometric blood pressure values in infants at 3, 6 and 12 months of age: a cohort study
Source: BMJ Paediatr Open. 2026 Jun 17;10(1):e004761. doi: 10.1136/bmjpo-2026-004761 (PMC13289193; doi:10.1136/bmjpo-2026-004761)
Supplement: online supplemental file 1 [file bmjpo-10-1-s001.pdf]

Supplemental Material

This appendix has been provided by the authors to give readers additional information about their work.

Supplement to: Vallevik EØ, Holten- Andersen MN, Lødrup Carlsen KC et al.
«Oscillometric blood pressure values in infants at 3, 6 and 12 months of age: a cohort study”

Table of contents

Standard Operating Procedures **2**
Blood pressure measurement 3 months 2
Blood pressure measurement 6 months 3
Blood pressure measurement 12 months 4
Supplemental tables **5**
Supplemental table 1. Anthropometrics at 3-, 6- and 12- month follow-up visits 5
Supplemental table 2. Comparison of blood pressure based on arousal state 6
Supplemental table 3. Comparison of various combinations of blood pressure readings 7

Standard Operating Procedure: Blood pressure measurement 3 months				
	Rev. No. 1	Date: 18.04.2016	Page **1** of **1**	

Purpose: To measure the blood pressure in infants 3 months of age, in the PreventADALL study.

Possible interferences: The baby has to be calm for a correct measurement. The cuff has to be of the right size. Too big cuff gives a measurement that is too low. Too small cuff gives a measurement that is slightly higher, as well as risking to cause venous congestion and discoloration of the limb.

Equipment:

Automatic oscillometric blood pressure monitor. GE Carescape Dinamap V100
Critikon 2525 Neonatal number 5, 8-15 cm blood pressure cuff.

Personnel qualifications: Trained study personnel.

Safety considerations: Do not place the cuff on an arm with potentially compromised circulation or on non-intact or injured skin.

Procedure:

- To find the right cuff size: Measure the infant's upper arm circumference by placing a measuring tape around the midpoint of the arm. Compare to the circumference ranges marked on the cuff. Avoid using a cuff with a width that extends over a joint.
- Squeeze all air from the cuff before placing it on the infant's right upper arm. The cuff should be wrapped snugly around the arm, not too tight and not too loose.
- Measurements should be done with the infant in a supine position on the mat with the right arm extended at the elbow joint. The child's arm should if possible be relaxed and resting on the mat, palm facing upward. This could be achieved by placing the arm along the body and using distractions, and if necessary by the examinator gently holding the child's hand still. Grasping of the hand and forceful holding of the arm should be avoided. After inflating the cuff, the monitor begins to deflate it and measures systolic pressure, MAP, and diastolic pressure.
- When the diastolic pressure has been determined, the monitor finishes deflating the cuff and posts the values to a display.
- Three measurements are made, with the cuff on the infant's right upper arm, and from each measurement blood pressure (systolic/diastolic), MAP and pulse rate is noted.
- The ambition is to register three measurements as similar as possible with the baby being in the same state, preferably calm. If one of the measurements deviates more than 20 mmHg in systolic pressure the blood pressure measurement is repeated until three measurements are within a range of 20 mmHg in systolic pressure.
- If three measurements within a range of 20 mmHg are not obtained after three additional measurements note the first three results.
- It must be noted if the infant is uneasy (struggling/moving/crying) during every single measurement, or if it is lying calm.

Purpose: To measure the blood pressure in infants 6 months of age, in the PreventADALL study.

Possible interferences: The baby has to be calm for a correct measurement. The cuff has to be of the right size. Too big cuff gives a measurement that is too low. Too small cuff gives a measurement that is slightly higher, as well as risking to cause venous congestion and discoloration of the limb.

Equipment:

Automatic oscillometric blood pressure monitor. GE Carescape Dinamap V100
Critikon 2525 Neonatal number 5, 8-15 cm blood pressure cuff.

Personnel qualifications: Trained study personnel.

Safety considerations: Do not place the cuff on an arm with potentially compromised circulation or on non-intact or injured skin.

Procedure:

- To find the right cuff size: Measure the infant's upper arm circumference by placing a measuring tape around the midpoint of the arm. Compare to the circumference ranges marked on the cuff. Avoid using a cuff with a width that extends over a joint.
- Note the circumference of the right upper arm.
- Squeeze all air from the cuff before placing it on the infant's right upper arm. The cuff should be wrapped snugly around the arm, not too tight and not too loose.
- Measurements should be done with the infant in a supine position on the mat with the right arm extended at the elbow joint. The child's arm should if possible be relaxed and resting on the mat, palm facing upward. This could be achieved by placing the arm along the body and using distractions, and if necessary by the examinator gently holding the child's hand still. Grasping of the hand and forceful holding of the arm should be avoided. After inflating the cuff, the monitor begins to deflate it and measures systolic pressure, MAP, and diastolic pressure.
- When the diastolic pressure has been determined, the monitor finishes deflating the cuff and posts the values to a display.
- Three measurements are made, with the cuff on the infant's right upper arm, and from each measurement blood pressure (systolic/diastolic), MAP and pulse rate is noted.
- The ambition is to register three measurements as similar as possible with the baby being in the same state, preferably calm. If one of the measurements deviates more than 20 mmHg in systolic pressure the blood pressure measurement is repeated until three measurements are within a range of 20 mmHg in systolic pressure.
- If three measurements within a range of 20 mmHg are not obtained after three additional measurements note the first three results.
- It must be noted if the infant is uneasy (struggling/moving/crying) during every single measurement, or if it is lying calm.

Standard Operating Procedure: Blood pressure measurement 12 months				
	Rev. No.	Date: 18.04.2016	Page **1** of **1**	

Purpose: To measure the blood pressure in infants 12 months of age, in the PreventADALL study.

Possible interferences: The child has to be calm for a correct measurement. The cuff has to be of the right size. A cuff that is too big gives a measurement that is too low. A cuff that is too small gives a measurement that is slightly higher, as well as risking to cause venous congestion and discoloration of the limb.

Equipment:

Automatic oscillometric blood pressure monitor. GE Carescape Dinamap V100.
Critikon 2525 Neonatal number 5, 8-15 cm blood pressure cuff.
Critikon Dura-Cuf Ref 2201, 12-19 cm blood pressure cuff.

Personnel qualifications: Trained study personnel.

Safety considerations: Do not place the cuff on an arm with potentially compromised circulation or on non-intact or injured skin.

Procedure:

- To find the right cuff size: Measure the infant's upper arm circumference by placing a measuring tape around the midpoint of the arm. Compare to the circumference ranges marked on the cuff. Avoid using a cuff with a width that extends over a joint.
- Note the size of the cuff.
- Squeeze all air from the cuff before placing it on the infant's right upper arm. The cuff should be wrapped snugly around the arm, not too tight and not too loose.
- Measurements should be done with the infant in a seated position (on caretakers lap) with the right arm extended at the elbow joint, palm facing upward. This could be achieved by placing the arm along the body and using distractions, and if necessary by the examiner gently holding the child’s hand still. Grasping of the hand and forceful holding of the arm should be avoided. After inflating the cuff, the monitor begins to deflate it and measures systolic pressure, MAP, and diastolic pressure.
- When the diastolic pressure has been determined, the monitor finishes deflating the cuff and posts the values to a display.
- Three measurements are made, with the cuff on the infant’s right upper arm, and from each measurement blood pressure (systolic/diastolic), MAP and pulse rate is noted.
- The ambition is to register three measurements as similar as possible with the baby being in the same state, preferably calm. If one of the measurements deviates more than 20 mmHg in systolic pressure the blood pressure measurement is repeated until, if possible, three measurements are within a range of 20 mmHg in systolic pressure.
- If three measurements within a range of 20 mmHg are not obtained after three additional measurements note the three results with the highest correspondence.
- Note down the state of the child for each measurement, either uneasy (struggling/moving/crying) or calm.

Supplemental tables

Supplemental table 1. Anthropometrics at 3-, 6- and 12-month follow-up visits.

Age, weight and length at each follow-up visit for infants with and without successful blood pressure measurements*, among those with at least one blood pressure reading available at that visit.

	Infants with successful measurements		Infants without successful measurements	
	**Boys**	**Girls**	**Boys**	**Girls**
	**3 months (N = 1646)**			
N	553 (55)	454 (45)	333 (52)	306 (48)
Age, months	3.1 (0.2)	3.1 (0.3)	3.1 (0.2)	3.1 (0.3)
Weight, kg	6.5 (0.7)	5.9 (0.7)	6.5 (0.7)	6.0 (0.7)
Length, cm	63 (2)	61 (2)	63 (2)	61 (2)
	**6 months (N = 1747)**			
N	655 (53)	578 (47)	256 (50)	258 (50)
Age, months	6.3 (0.5)	6.2 (0.4)	6.2 (0.4)	6.2 (0.4)
Weight, kg	8.4 (0.9)	7.7 (0.8)	8.4 (1.0)	7.8 (0.9)
Length, cm	70 (2)	68 (2)	69 (2)	68 (2)
	**12 months (N = 1304)**			
N	451 (56)	357 (44)	261 (53)	235 (47)
Age, months	12.5 (0.7)	12.5 (0.7)	12.5 (0.8)	12.4 (0.7)
Weight, kg	10.4 (1.1)	9.7 (1.0)	10.4 (1.1)	9.8 (1.0)
Length, cm	77 (3)	76 (3)	77 (3)	75 (3)

Data are n (%) or mean (SD). Percentages may not sum to 100 because of rounding
* A successful measurement is defined by three available blood pressure readings obtained in a calm state and a systolic blood pressure range of maximum 20 mmHg.

Supplemental table 2. Comparison of blood pressure based on arousal state.

Comparison of systolic, diastolic and mean arterial blood pressure at 3, 6 and 12 months of age, based on arousal state. Includes infants who had at least one blood pressure reading in each state, calm and agitated, at each age. P-values are determined by paired t-tests comparing the mean blood pressure during calm and agitated states at each age.

	**3 months (N = 79)**		**6 months (N = 150)**		**12 months (N = 139)**	
	**Calm**	**Agitated**	**Calm**	**Agitated**	**Calm**	**Agitated**
**Systolic blood pressure, mmHg**						
Mean (SD)	95 (9)	105 (13)	98 (8)	109 (13)	97 (9)	105 (12)
Mean difference* (95% CI)	9.9 (6.7-13.0)		11.1 (9.2-12.9)		7.8 (5.8-9.9)	
P-value	< 0.001		< 0.001		<0.001	
p5	83	86	85	92	85	86
p50	95	106	98	107	96	104
p95	114	129	114	135	112	126
**Diastolic blood pressure, mmHg**						
Mean (SD)	57 (9)	65 (11)	59 (8)	65 (10)	59 (9)	62 (10)
Mean difference* (95%CI)	8.6 (5.6-11.6)		5.8 (4.1-7.4)		3.2 (1.5-4.8)	
P- value	< 0.001		< 0.001		<0.001	
p5	43	46	44	49	42	44
p50	57	65	59	63	60	62
p95	71	86	72	82	73	79
**Mean arterial pressure, mmHg**						
Mean (SD)	70 (8)	79 (11)	72 (7)	79 (9)	72 (8)	77 (10)
Mean difference* (95%CI)	9.3 (6.7-12.1)		6.6 (5.1-8.2)		4.7 (3.2-6.2)	
P- value	< 0.001		< 0.001		< 0.001	
p5	55	63	62	66	60	58
p50	69	78	72	79	71	77
p95	82	98	84	97	86	94

p, percentile.
*Mean difference is the mean of the difference between the calm and the agitated readings.

Supplemental table 3. Comparison of various combinations of blood pressure readings.

Descriptive comparison of the mean, SD and percentiles for various combinations of three calm readings at 3, 6 and 12 months of age: the mean of three readings (1), the mean of the two closest readings (2), the mean of the first two readings (3), the mean of the last two readings (4) and the first reading alone (5).

	3 months (N = 1007)					6 months (N = 1233)					12 months (N = 808)				
**Combination of readings**	**1**	**2**	**3**	**4**	**5**	**1**	**2**	**3**	**4**	**5**	**1**	**2**	**3**	**4**	**5**
**Systolic blood pressure, mmHg**															
Mean (SD)	95 (8)	95 (9)	95 (8)	95 (8)	95 (9)	97 (8)	97 (8)	97 (8)	97 (8)	97 (9)	96 (8)	95 (8)	96 (8)	96 (8)	96 (9)
p5	84	82	83	83	82	86	85	86	85	85	84	83	83	84	82
p50	95	95	95	95	94	96	96	97	97	96	95	95	95	95	95
p95	110	111	111	110	113	111	112	113	112	115	110	111	111	110	113
Mean (SD) of difference*	9 (5)	2 (2)	6 (5)	5 (4)	N/A	8 (5)	2 (2)	5 (4)	5 (4)	N/A	8 (5)	2 (2)	5 (4)	5 (4)	N/A
**Diastolic blood pressure, mmHg**															
Mean (SD)	56 (7)	56 (8)	57 (8)	55 (8)	58 (9)	57 (7)	57 (8)	58 (7)	57 (8)	59 (8)	57 (7)	57 (8)	58 (7)	57 (7)	58 (8)
p5	44	42	44	42	44	47	45	47	45	46	45	45	46	45	45
p50	56	56	57	55	57	57	57	58	57	58	57	58	58	57	58
p95	69	70	70	70	73	69	71	70	70	72	69	70	70	69	71
Mean (SD) of difference*	11 (7)	3 (2)	7 (6)	7 (6)	N/A	9 (7)	2 (2)	6 (5)	5 (5)	N/A	9 (6)	2 (2)	5 (5)	5 (5)	N/A
**Mean arterial pressure, mmHg**															
Mean (SD)	69 (7)	69 (8)	70 (7)	69 (8)	70 (8)†	72 (6)	72 (7)	72 (6)	71 (7)	72 (7)†	71 (7)	71 (7)	71 (7)	71 (7)	71 (8)†
p5	59	57	59	57	59	62	61	62	61	62	60	60	60	60	59
p50	69	69	69	69	70	72	71	72	71	72	71	71	71	71	71
p95	82	83	83	82	85	82	83	83	83	83	83	83	83	83	84
Mean (SD) of difference*	8 (6)	2 (2)	5 (5)	5 (5)	N/A	7 (5)	2 (2)	4 (4)	4 (4)	N/A	7 (5)	2 (2)	4 (4)	4 (4)	N/A

p, percentile.
*Difference between the (max and min) measurements.
†N(mean arterial pressure, 3 months) = 1005. N(mean arterial pressure, 6 months) = 1231. N(mean arterial pressure, 12 months) = 806
