# Supplementary material for: Oscillometric blood pressure values in infants at 3, 6 and 12 months of age: a cohort study
Source: BMJ Paediatr Open. 2026 Jun 17;10(1):e004761. doi: 10.1136/bmjpo-2026-004761 (PMC13289193; doi:10.1136/bmjpo-2026-004761)
Supplement: Supplementary file 2 [file bmjpo-2026-004761.reviewer_comments.pdf]

PEER REVIEW HISTORY

BMJ Paediatrics Open publishes all reviews undertaken for accepted manuscripts. Reviewers are asked to complete a checklist review form and are provided with free text boxes to elaborate on their assessment. These free text comments are reproduced below.

ARTICLE DETAILS

**TITLE (PROVISIONAL)**	Oscillometric blood pressure values in infants at 3,6 and 12 months of age: a cohort study
**AUTHORS**	Ørbeck Vallevik, Elin<br>Holten-Andersen, Mads Nikolaj<br>Carlsen, Karin Cecilie Lødrup<br>Skjerven, Håvard Ove<br>Vettukattil, Riyas<br>Rueegg, Corina Silvia<br>Rehbinder, Eva M<br>Nordlund, Björn<br>Söderhäll, Cilla<br>Hedlin, Gunilla<br>Haugen, Guttorm Nils<br>Staff, Anne Cathrine<br>Nilsen, Morten<br>Holmstrøm, Henrik

VERSION 1 – REVIEW

**REVIEWER NAME**	Nathan, Joanna
**REVIEWER AFFILIATION**	
**REVIEWER CONFLICT OF INTEREST**	No Conflict of Interest
**AI DISCLOSURE**	No, I have not used AI
**DATE REVIEW RETURNED**	23-Apr-2026

**GENERAL COMMENTS**	I thought this was a much needed and well-designed study that provides valuable normative data. And also the finding that a single reading in a calm infant is comparable to the mean of multiple readings is highly relevant for busy clinical settings.<br><br>It might be helpful to briefly mention in the intro why oscillometric devices have become the standard of care (e.g., ease of use, reduced observer bias) compared to auscultatory methods, further justifying the need for device-specific normative data.<br><br>I wasn't clear on why there was a change in positioning from supine (at 3 and 6 months) to sitting (at 12 months)? May be for clinician ease but probably best to explain. Particularly because a slight decline in BP at 12 months was attributed it to the change from supine to sitting position.

VERSION 1 – AUTHOR RESPONSE

Reviewer: 1
Joanna Nathan
Comments to the Author

I thought this was a much needed and well-designed study that provides valuable normative data. And also the finding that a single reading in a calm infant is comparable to the mean of multiple readings is highly relevant for busy clinical settings.

We thank the reviewer for her appreciation of our manuscript.

It might be helpful to briefly mention in the intro why oscillometric devices have become the standard of care (e.g., ease of use, reduced observer bias) compared to auscultatory methods, further justifying the need for device-specific normative data.

We thank the reviewer for this helpful suggestion and agree that this addition strengthens the rationale for the study. Accordingly, we have added the following sentence to the second paragraph of the Introduction (page 4): “..., as it is convenient and easy to use, with less dependence on the person performing the measurement compared to auscultatory methods.”

I wasn't clear on why there was a change in positioning from supine (at 3 and 6 months) to sitting (at 12 months)? May be for clinician ease but probably best to explain. Particularly because a slight decline in BP at 12 months was attributed it to the change from supine to sitting position.

We thank the reviewer for this comment, and for the opportunity to clarify the manuscript.

The change in positioning from supine at 3 and 6 months to sitting at 12 months reflects standard clinical practice and feasibility when measuring blood pressure in infants. While younger infants can be more easily measured in a supine position, older infants are often more difficult to keep still in this position, and measurements are therefore more reliably obtained while sitting.

We have clarified this in the Methods section, page 6, by adding the following sentence: “, reflecting standard clinical practice and feasibility at these ages”.
